# Cemented long versus standard femoral stem in proximal femoral metastasis: a noninferiority single-blinded quasi-randomized clinical trial

**DOI:** 10.1007/s00068-021-01875-x

**Published:** 2022-02-13

**Authors:** Ibrahim Mahmoud Abdelmonem, Sherif Ishak Azmy, Ayman Mohammad EL Masry, Ahmed k el ghazawy, Ahmed sayed kotb, Ayman Abdelaziz Bassiony

**Affiliations:** Department of Orthopaedic Surgery, Faculty of Medicine, Ain Shams University, Cairo, Egypt

**Keywords:** Pathological fracture, Hip fracture, Metastasis, Hip arthroplasty, Femoral stem

## Abstract

**Background and purpose:**

Proximal femur is a common site for metastasis, it has a significant impact on patient’s quality of life, and traditional treatment aims at protecting as much as possible from the femur. However, recent studies have demonstrated increased rate of complications and questioned the need for long stem in this high-risk group. Our purpose is to determine whether standard-length femoral stem is noninferior to long femoral stem in the treatment of proximal femoral metastasis.

**Patients and methods:**

Between 2019 and 2021, we prospectively included 24 patients with proximal femoral metastasis leading either to impending or pathological fractures (5 and 19 cases, respectively). We included patients with lesions due to metastasis, lymphoma, or multiple myeloma. Patients were quasi-randomized based on their order of presentation into two groups based on the femoral stem length, cemented standard (group 1) and long (group 2) femoral stem. Oncological complications, survival, stem complications, and functional outcomes were recorded and analyzed using SPSS 25.

**Results:**

24 patients were included in the final analysis, 13 case in group 1 and 11 in group 2, and mean age 57.6 years. Mean follow-up duration was 10 months, and 11 patients died of the whole-study population with mean survival of (10.85 ± 2.23, 8.82 ± 3.6) months in group 1, 2, respectively. The complication rate was higher in the standard group; however, this difference did not reach statistical significance. No difference was found between study groups regarding functional outcomes, except for VAS at 6 months which was higher in standard group.

**Conclusion:**

We believe that the ubiquitous use of long stem in the management of proximal femoral metastasis should be questioned considering the expected patient survival and low rate of complications associated with the use of standard stem.

**Clinicaltrials.gov registration number:**

NCT04660591.

## Introduction

Bone metastases are a major source of morbidity and mortality in patients with cancer. The proximal femur is the most common long bone to be affected and is the second most common site for skeletal metastasis, with the spine being the first. Within the proximal femur, about 50% of lesions occur in the femoral neck, 20% in the per-trochanteric region, and 30% in the subtrochanteric region. The breast, prostate, and lungs are the most common origin of proximal femoral metastases [1, 2].

Recent advances in cancer treatment have led to increased survival and life expectancy [3]. However, by the time metastatic bone disease is diagnosed, the prognosis of the patient is typically poor. Proximal femoral metastasis has a significant impact on the quality of life of affected patients, either due to pain from the lesion, impending fracture, or the inability to mobilize due to the fracture [4]. Limited mobility in this high-risk group may lead to further complications, such as bed sores and repeated infections; some complications, such as thromboembolic events, may be fatal [5].

The aim of treatment in these patients is early mobilization, with the fewest possible complications. Arthroplasty is the optimal treatment option in patients with proximal femoral metastasis as it provides the advantage of early mobilization and pain relief [6–8]. Different reconstruction options, such as multiple stem designs with different fixation methods, are available [3]. Intramedullary cementing is usually needed in such patients to provide adequate stability of the femoral stem. However, cementing is not devoid of complications; cement reaction and cardiopulmonary compromise are possible complications, the risk of which increases with increasing stem length [9, 10].

Using a long femoral stem has so far been the standard of care in proximal femoral metastases, as it is believed to protect the femur as much as possible from future distal lesions [11]. However, recent consensus published by the Musculoskeletal Tumor Society (MSTS) has recommended the use of standard-length femoral stems in patients with pathological femoral neck fractures [12]. As the incidence of developing new metastatic lesions requiring surgical treatment in the same femur during the lifetime of the patients with metastatic cancer is unclear, the advantages of a long femoral stem may only be hypothetical, but may instead be associated with an increased risk of complications; combining a long stem with cement further increases the possibility of complications, especially in patients with other preexisting medical comorbidities or poor bone quality from the metastatic disease [9, 11, 13].

It is therefore unclear if the additional stem length will add to the stability and decrease later fractures in patients with proximal femoral metastasis. We hypothesize that the standard-length femoral stem is not inferior to the long femoral stem regarding stem-related complications, and that its use is associated with less perioperative morbidity and mortality while providing enough stability but without compromising the functional and oncological outcomes. The aim of this study was to compare the oncological complications, including the survival and functional outcomes of different stem lengths in patients with proximal femoral metastasis.

## Methods

### Study design

The study was designed as a single-center, single-blinded, parallel-group clinical trial. Patients were enrolled if they presented with proximal femoral metastases to our institution between November 2019 and March 2020. Due to the small sample size, only the first participant was randomized; further allocation was sequential in a 1:1 ratio in each study group based on the order of presentation. The physicians allocated to both intervention groups were aware of the allocated arm, whereas the patients were kept blinded to the allocation.

The primary objective was a noninferiority comparison between the standard and long femoral stems on the incidence of stem-related complications (fracture distal to the stem, infection, or dislocation). Survival, perioperative, and postoperative complications (mortality, cardiopulmonary events, intraoperative bleeding, and duration of hospital stay) were also recorded.

Using data from a previous study [11], we calculated the sample size needed by setting the type 1 error *α* at 0.05. A sample size of 11 in each group was calculated to achieve 80% power to detect a noninferiority margin difference of 8%, assuming a complication rate of 2% in the long-stem group and 10% in the standard-stem group.

### Inclusion criteria

All patients older than 18 years with either proximal femoral metastases or proximal femoral lesions due to multiple myeloma or lymphoma, presenting with an impending or actual pathological fracture, were included in our study. An impending fracture was defined as any painful lesion with more than 50% cortical destruction determined by plain X-rays. All patients with lesions in the femoral head, neck, and intertrochanteric and per-trochanteric regions were included.

### Exclusion criteria

We excluded patients with an expected survival of less than 4 weeks and patients who had undergone previous ipsilateral hip surgery and fractures due to metabolic disease. Lesions affecting the subtrochanteric region, lesions due to primary benign or malignant bone tumor, and lesions requiring proximal femoral resection and reconstruction with mega-prostheses were also excluded.

### Preoperative evaluation

Preoperative clinical and radiological evaluation of the patients included documentation of the complete medical history, full physical examination, and CT imaging of the chest, abdomen, and pelvis. A bone scan was also obtained to detect the number of skeletal metastases. Assessment of fitness for surgery, expected survival, and oncological and functional outcomes, including the MSTS scoring system, visual analog scale (VAS) pain score, the Eastern cooperative oncology group (ECOG) scale of performance status, the Karnofsky performance status score, and health-related quality of life (HRQOL) score, were also recorded.

### Surgical procedure

Patients were prepared for the surgery with a complete preoperative evaluation and anesthesia consultation.

The acetabular or femoral components of the implant were chosen by the operating surgeon based on the intraoperative assessment. The choice of the femoral implant included a cemented Biomet CPT^®^ 12/14 130 mm standard-length femoral stem hip system for Group 1 and a Biomet CPT^®^ 12/14 long-stem hip system for Group 2. All stems were manufactured by Biomet Inc. (Warsaw, IN, USA). None of the included patients had acetabular involvement; the choice of the acetabular component included total hip replacement, tripolar hip arthroplasty, or hemiarthroplasty, based on the patient’s level of activity and presence of arthritic changes in the acetabulum. A modified Hardinge approach was utilized. The excised specimen including the femoral head and neck was sent for histopathological assessment. Furthermore, a drain was inserted in all the patients.

### Postoperative follow-up

All patients were followed up daily, and wound soaking, drain amount, and type of discharge were assessed. The first postoperative dressing change was undertaken on day 2, and a second dressing change on day 4. All patients were encouraged to start weight bearing as soon as possible. Patients were discharged on oral anticoagulant and analgesic. Postoperative adjuvant treatment was planned according to the primary cancer type using a multidisciplinary team from the oncology, radiotherapy, and physiotherapy departments. In our study, two patients received local preoperative radiotherapy and 14 patients received postoperative radiotherapy. Patients were followed up in the outpatient clinic 1 month postoperation and subsequently every 3 months. Postoperative complications and functional scores, as described above, were recorded.

### Statistical analysis

A *p* value of less than 0.05 was used to determine statistical significance. Data were collected using Google forms, and the results were exported into an Excel spreadsheet. The collected data were revised, coded, tabulated, and entered into a PC using Statistical Package for Social Science (SPSS^®^ 25). The mean, standard deviation, and range for parametric numerical data were used; non-numerical data were reported as frequency and percentage. Furthermore, Student’s *t* test was used to assess the statistical significance of the difference between the two means. The Mann–Whitney *U* test was used for non-parametric data. The Chi-squared test and Fisher’s exact test were used to examine the relationship between two qualitative variables. Patient survival was presented on a Kaplan–Meier curve and was estimated using the log-rank test.

## Results

### Demographic data

**Fig. 1:**
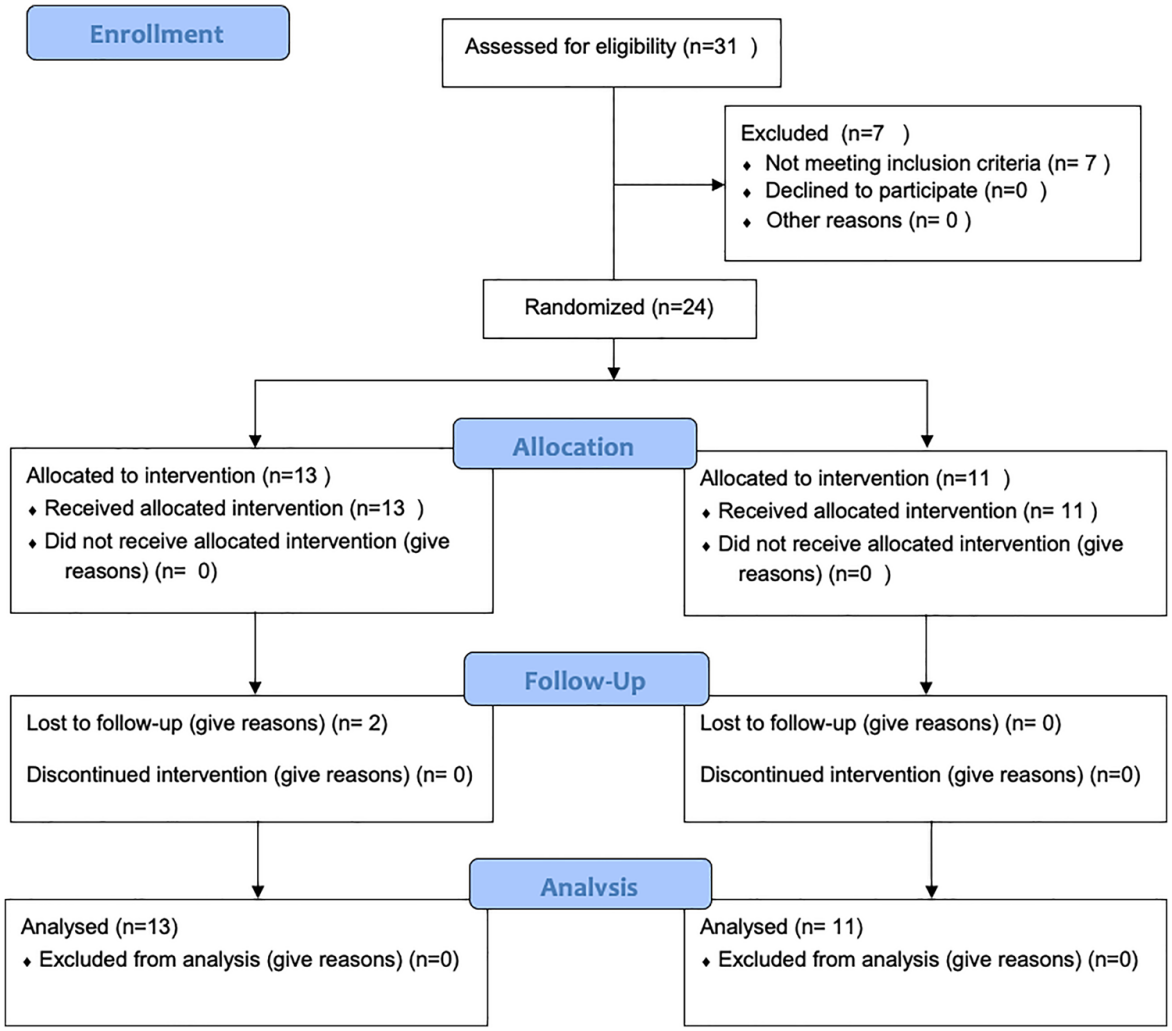
Participant flow diagram according to consolidated standards of reporting trial criteria

Twenty-four patients were included in the clinical trial, 13 in Group 1 and 11 in Group 2. The mean age was 57.5 ± 13.6 years (Fig. 1). Duration of follow-up ranged between 1 and 12 months in both groups, with a mean duration of 10 ± 3 months. The preoperative general and oncological status (demographics, primary cancer type, fracture site, and functional status) of the included patients are reported in Table 1. Time from fracture diagnosis to surgical treatment was 14.4 ± 9.9 days.

**Table 1:** Study groups basic demographics and preoperative data

	Study group		Test of significance		
	Standard	Long			
	Mean ± SD *N* (%)	Mean ± SD *N* (%)	Value	*p* Value	Sig
Age	53.85 ± 13.64	62 ± 12.87	*t* = − 1.497	0.149^(T)^	NS
Gender					
Male	5 (38.46%)	6 (54.55%)	*X*^2^ = 0.621	0.431^(C)^	NS
Female	8 (61.54%)	5 (45.45%)			
Fracture site					
Neck	9 (69.23%)	7 (63.64%)		1.00^(F)^	NS
Intertrochanteric	2 (15.38%)	2 (18.18%)			
Multiple	2 (15.38%)	2 (18.18%)			
Fracture status					
Impending	3 (23.08%)	2 (18.18%)		1.00^(F)^	NS
actual fracture	10 (76.92%)	9 (81.82%)			
Time from diagnosis of fracture to surgery(days)	11 (7–26)	9 (6–21)	*U* = 61.0	0.542^(M)^	NS
Primary cancer type					
Unknown	2 (15.38%)	2 (18.18%)		0.921^(F)^	NS
Breast	6 (46.15%)	4 (36.36%)			
Multiple myeloma	2 (15.38%)	1 (9.09%)			
Prostate	2 (15.38%)	1 (9.09%)			
Renal	1 (7.69%)	0 (0%)			
Lung	0 (0%)	1 (9.09%)			
Colon	0 (0%)	1 (9.09%)			
Adenocarcinoma	0 (0%)	1 (9.09%)			
Number of skeletal metastasis	2 (1–3)	2 (2–3)	*U* = 60.5	0.504^(M)^	NS
Preoperative radiotherapy					
No	12 (92.31%)	10 (90.91%)		1.00^(F)^	NS
Yes	1 (7.69%)	1 (9.09%)			
Postoperative radiotherapy					
No	6 (46.15%)	4 (36.36%)		0.697^(F)^	NS
Yes	7 (53.85%)	7 (63.64%)			

^(F)^Fisher’s exact test of significance

^(M)^Mann–Whitney test of significance (*U* = Mann–Whitney test value)

All patients except one had unilateral disease; the patient with bilateral disease (case 8) only received radiotherapy on the second side as they declined to undergo another operation (Table 2). One case from each study group (cases 3 and 14) did not undergo follow-up at 8 and 10 months postoperatively.

**Table 2:** Demographic data, management option, and outcomes of study participants

Case ID	Group	Age	Gender	Primary cancer	Fracture site	Fracture status	No. of Sk metastasis	Mod Katigiri	Implant	Time to walk (days)	Mortality time (months)	Complications
1	Standard	45	Female	Breast	Neck	Fracture	2	3	Bipolar	7	7	Mortality
2	Long	58	Male	Adenocarcinoma	Neck	Fracture	1	4	Bipolar	1	5	Mortality (COVID)
3	Standard	43	Male	Renal	Neck	Impending	1	3	THA	2	–	
4	Long	87	Female	Breast	Intertroch	Fracture	1	3	Bipolar	2	7	Mortality
5	Standard	49	Female	UNKNOWN	Intertroch	Fracture	1	5	Bipolar	8	12	Mortality
6	Long	37	Female	Breast	Multiple	Impending	3	5	Bipolar	21	1	Mortality
7	Standard	27	Female	Breast	Neck	Fracture	5	4	Tripolar	14	–	
8	Long	55	Female	COLON	Neck	Fracture	2	4	THa	7	–	
9	Standard	55	Female	Breast	Neck	Impending	1	2	Tripolar	3	–	
10	Long	73	Male	Prostate	Neck	Fracture	2	4	Tripolar	30	–	
11	Standard	75	Female	Breast	Neck	Fracture	1	2	Tripolar	30	–	
12	Long	65	Male	Lung	Neck	Fracture	2	6	Bipolar	20	10	Mortality
13	Standard	67	Male	Prostate	Neck	Fracture	2	2	Tripolar	2	–	
14	Long	70	Male	MM	Intertroch	Fracture	6	3	THA	31	–	
15	Standard	36	Female	MM	Neck	Fracture	6	1	THA	6	8	Lost to FUP
16	Long	59	Female	Breast	Neck	Fracture	2	3	Bipolar	21	–	
17	Standard	59	Female	Breast	Neck	Fracture	1	3	Bipolar	28	8	Mortality
18	Long	61	Male	Unknown	Neck	Fracture	2	1	Bipolar	41	7	Mortality
19	Standard	66	Male	MM	Intertroch	Fracture	3	3	Tripolar	19	11	Infection, lost to FUP
20	Long	51	Female	Breast	Neck	Impending	3	2	THA	27	–	
21	Standard	55	Female	Breast	Multiple	Impending	2	2	Bipolar	32	–	
22	Long	66	Male	Unknown	Multiple	Fracture	2	3	Bipolar	26	8	Mortality
23	Standard	56	Male	Unknown	Neck	Fracture	1	1	THA	12	12	Mortality
24	Standard	67	Male	Prostate	Multiple	Fracture	5	4	THA	30	6	Infection, dislocation, mortality

*MM* multiple myeloma, *THA* conventional total hip arthroplasty, *FUP* follow-up

### Survival

The mean survival for the whole-study population was 9.95 ± 0.64 months. We found no significant difference between Group 1 and Group 2, with overlapping 95% confidence intervals of 10.85 ± 0.66 and 8.9 ± 1.06 months, respectively (Table 3).

**Table 3:** Mean survival of both treatment groups with confidence interval

Group	Mean			
	Estimate	SE	95% Confidence interval	
			Lower bound	Upper bound
Standard	10.846	0.664	9.544	12.149
Long	8.909	1.056	6.840	10.979
Overall	9.953	0.644	8.691	11.214

**Fig. 2:**
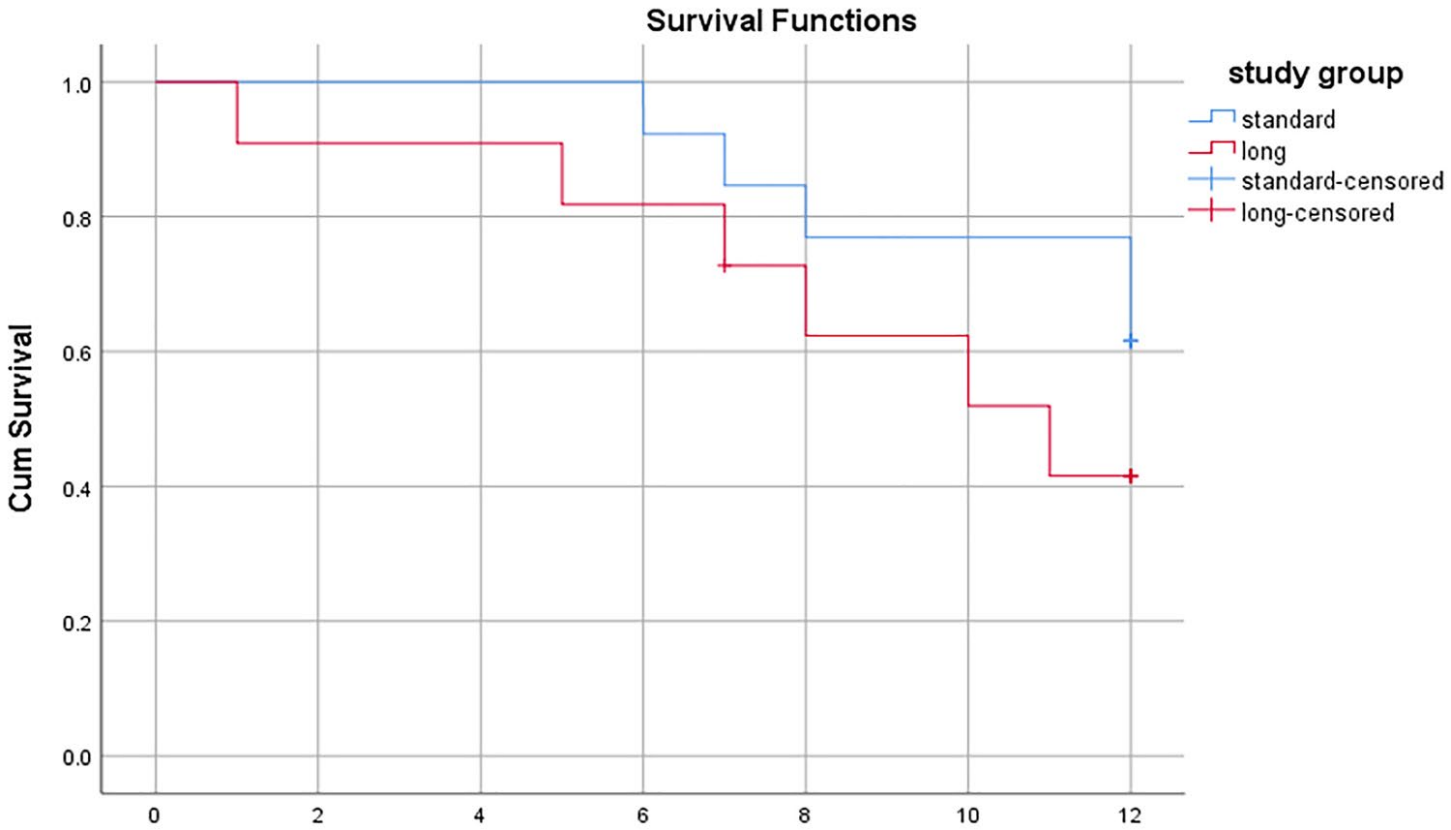
Kaplan–Meier survival analysis curve of both treatment groups

Survival data were calculated and presented using a Kaplan–Meier curve (Fig. 2). One-year overall survival for the whole-study population from the time of diagnosis of the fracture was 54%, with survival of 61.5% and 45.5% for Groups 1 and 2, respectively. Of the 11 reported mortality cases, breast cancer was the primary source in four cases. Three patients had lesions in more than one region, and one case had an impending fracture. Nine cases underwent hemiarthroplasty and two cases underwent conventional total hip replacement. We found no statistically significant correlation between the number of skeletal metastases at the time of surgery and survival.

One patient (case 6) with multiple metastases, including a pathological midshaft humerus fracture and an impending pathological femoral fracture, died on postoperative day 14. He had undergone a two-stage operation, with debulking and fixation using an intramedullary rush pin nail. Plating and cementing of the humeral fracture were the first stage and hemiarthroplasty with a cemented long femoral stem was the second stage of the operation. The patient died 2 days after the second operation in the intensive care unit.

### Functional outcomes

All patients included in the study reported significant improvement in quality of life (HRQOL) and pain (VAS) scores compared to preoperative status (Table 4). However, there were no significant differences in the postoperative functional (ECOG, HRQOL, and MSTS) and pain (VAS) scores between the two groups. The only significant difference was a higher VAS score 6 months postoperation in the standard-stem group (1.31 ± 0.48) compared to the long-stem group (1 ± 0) (*p* = 0.04).

**Table 4:** Pre- and postoperative MSTS and VAS scores of both treatment groups

	Study group		Student’s *t* test		
	Standard	Long			
	Mean ± SD	Mean ± SD	*t*	*p* Value	Sig
Preoperative MSTS	6.75 ± 1.36	9	− 1.593	0.139	NS
MSTS 1 month	20.5 ± 2.24	19	0.645	0.532	NS
MSTS 6 months	23.33 ± 2.02	21	1.113	0.290	NS
preoperative VAS	6.69 ± 1.8	6.91 ± 1.38	− 0.327	0.747	NS
Postoperative VAS 1 month	1.62 ± 0.77	1.2 ± 0.42	1.653	0.114	NS
Postoperative VAS 6 months	1.31 ± 0.48	1 ± 0	2.309	0.040	S
Time (days) to mobilization^a^	12 (6—28)	21 (7–30)	*U* = 56.5	0.384 (M)	NS

^(T)^Student’s *t* test of significance (*t* = Student’s *t* test value)

^a^Defined as independent walking with or without walking aids

Patients in Group 2 had a higher average time to achieve independent mobilization (21 days) compared to Group 1 (12 days); however, this difference did not reach statistical significance.

### Complications and perioperative outcomes

We found no significant differences between the two groups in operative time, duration of hospital stay, or need for blood transfusion. We found a very low rate of adverse events overall in the whole-study group. We did not face any intraoperative complications; however, we came across a total of only three postoperative complications (two infections and one stem displacement) in two patients in the standard-stem group (Table 2). One patient with a pathological fracture due to multiple myeloma had a superficial wound infection that was managed conservatively. The other case underwent revision for stem displacement, requiring operative relocation, and recementing. This patient subsequently developed a periprosthetic joint infection that failed to resolve by debridement, and which necessitated the removal of the implant. There were no cardiopulmonary complications or fractures distal to the stem in either group.

## Discussion

The proximal femur is the most common site for long-bone metastases, and due to the biomechanical nature of this site, surgery is often mandatory [1]. The primary aim of surgical intervention is to provide mobility as soon as possible. Management options include fixation using an intramedullary device, standard endoprosthetic replacement, or modular endoprosthesis [6, 7].

Advantages of arthroplasty over fixation include earlier mobility, improved durability, and fewer postoperative complications [7, 8]. However, arthroplasty is associated with more healthcare costs and morbidity, and is therefore better reserved for patients with longer expected survival.

Our findings suggest that stem length has no statistically significant effect on functional or oncological outcomes or complications, when used in the management of proximal femoral metastasis.

We found low complication rates overall in the whole-study population. Our study was powered to detect noninferiority rather than superiority of standard femoral stem outcomes compared to long femoral stem. The low rate of complications could not be attributed to stem length; however, we believe that it is mainly due to the decreased patient survival following the diagnosis of metastatic bone disease. Both groups had significant postoperative improvement in overall quality of life and pain scores at different time points with no significant difference between the groups. The standard-stem group had a higher reoperation rate related to nononcologic causes and a lower mortality rate; however, these differences did not reach statistical significance.

One of the established principles in the management of long-bone metastasis is using long fixation devices to protect the entire bone from future lesions [14]. However, in cases of cemented arthroplasty, the use of a long femoral stem will not only have a higher cost but will also predispose this high-risk patient category to more complications that could be fatal, such as cardiopulmonary events and death [9, 13, 15].

Several studies have challenged this concept, and have questioned the need for protecting the femoral neck from future lesions in cases of fixation for pathological diaphyseal femoral fractures. Moon et al. reported no newly developed femoral neck lesions after intramedullary nailing of a cohort of 145 patients with femoral diaphyseal lesions [16]. Another study by Alvi and Damron reported disease progression and development of new lesions following intramedullary fixation and long-stem arthroplasty, and concluded that the risk associated with using longer implants is considerably higher than the complications attributed to disease progression and development of new lesions [14].

In our study, we had no cases of newly developed distal lesions or progression of the already present femoral lesion, which is the main hypothesis of longer stem use. We believe that the low complication rate and satisfactory improvement in functional outcomes is due to the limited survival and functional demands in this selected patient population. The prognosis of patients with proximal femoral metastasis is considered generally poor, with most studies reporting a 1-year survival rate of 50% regardless of management. Our 1-year survival rate was 54% (11/24), which is comparable to that reported in the literature. Other studies reported even lower 1-year survival rate, reaching as low as 17% in a cohort of 139 patients with proximal femoral metastasis managed by different surgical methods [17]. A study by Selek et al. reported a 27% 1-year survival rate in a cohort of patients with proximal femoral metastasis treated with endoprosthetic reconstruction [18]. We believe that in this select patient population, due to a short patient survival time and less functional demands, the implant usually outlives the patient.

The evidence addressing the use of long femoral stems in proximal femoral metastases is scarce and not supported by high-quality comparative studies. Clinical practice guidelines by the MSTS found no reliable evidence supporting the use of a long femoral stem, and their consensus is to avoid its routine use if no distal lesions are present at the time of diagnosis [12].

A study by Randall et al. focused on cement-related cardiopulmonary complications. In a study of 29 long femoral stems, they attempted to minimize cement-related complications using techniques, such as slow controlled stem insertion, suctioning during cementing, and the use of early low-viscosity polymethylmethacrylate. They reported cement-related hypotension in 14% cases and a worsening of mental status postoperatively in 3% cases [9]. Another study by Peterson et al. found good functional outcomes along with a low complication rate associated with the use of a long femoral stem [15].

The only study comparing different femoral stem lengths in cases of proximal femoral metastases found a low incidence of complications overall in the whole-study group and no correlation between stem length and complication rate [11]. The study retrospectively included 203 patients, and divided into three groups according to the stem length (long, medium, and short). The authors reported 11 cases of local tumor progression, out of which only three cases underwent revision, two cases underwent revision due to nononcological causes, and five cases developed new distal lesions. None of these complications could be correlated to stem length. The authors reported higher cardiopulmonary complication rates in the long-stem group compared to the other two groups combined (18% versus 7.5%, respectively). The study was limited by its retrospective design, heterogeneous sample, and a long study duration that may have exposed the study population to different adjuvant treatment protocols, stem designs, and operating surgeons.

To our knowledge, our study is the first prospective study comparing standard and long femoral stems in patients with proximal femoral metastases, multiple myeloma, and lymphoma. We attempted to eliminate the confounding factors associated with the previous studies by using a comparative, prospective study design, similar stem design, and same operating surgeons.

Our study has several limitations. These include the absence of allocation concealment and a short follow-up period. The study was performed in a single center in a developing country; this center treats patients mainly from low socioeconomic classes, this could limit the application of study results on whole population. We designed our study and calculated the sample size to detect inferiority of the standard stem if present; however, some effects may have been missed if it was less than the lower margin of effects used to calculate the sample size.

We attempted to overcome the problems associated with the small sample size using the noninferiority hypothesis to detect a difference in complication rate between the two groups. We also tried to minimize confounding variables by prospectively collecting data from all patients. In addition, all operations were undertaken in a short period of time during which the surgical team and instruments did not change.

## Conclusion

Based on the findings of this study, we believe that the ubiquitous use of long femoral stems in the management of proximal femoral metastasis should be questioned, considering the expected patient survival and low rate of complications associated with the use of standard stems.
